# Visual attention for linguistic and non-linguistic body actions in non-signing and native signing children

**DOI:** 10.3389/fpsyg.2022.951057

**Published:** 2022-09-09

**Authors:** Rain G. Bosworth, So One Hwang, David P. Corina

**Affiliations:** ^1^NTID PLAY Lab, National Technical Institute for the Deaf, Rochester Institute of Technology, Rochester, NY, United States; ^2^Center for Research in Language, University of California, San Diego, San Diego, CA, United States; ^3^Center for Mind and Brain, University of California, Davis, Davis, CA, United States

**Keywords:** visual attention, eye tracking, infants, children, sign language, gestures, pantomime, body actions

## Abstract

Evidence from adult studies of deaf signers supports the dissociation between neural systems involved in processing visual linguistic and non-linguistic body actions. The question of how and when this specialization arises is poorly understood. Visual attention to these forms is likely to change with age and be affected by prior language experience. The present study used eye-tracking methodology with infants and children as they freely viewed alternating video sequences of lexical American sign language (ASL) signs and non-linguistic body actions (self-directed grooming action and object-directed pantomime). In Experiment 1, we quantified fixation patterns using an area of interest (AOI) approach and calculated face preference index (FPI) values to assess the developmental differences between 6 and 11-month-old hearing infants. Both groups were from monolingual English-speaking homes with no prior exposure to sign language. Six-month-olds attended the signer’s face for grooming; but for mimes and signs, they were drawn to attend to the “articulatory space” where the hands and arms primarily fall. Eleven-month-olds, on the other hand, showed a similar attention to the face for all body action types. We interpret this to reflect an early visual language sensitivity that diminishes with age, just before the child’s first birthday. In Experiment 2, we contrasted 18 hearing monolingual English-speaking children (mean age of 4.8 years) vs. 13 hearing children of deaf adults (CODAs; mean age of 5.7 years) whose primary language at home was ASL. Native signing children had a significantly greater face attentional bias than non-signing children for ASL signs, but not for grooming and mimes. The differences in the visual attention patterns that are contingent on age (in infants) and language experience (in children) may be related to both linguistic specialization over time and the emerging awareness of communicative gestural acts.

## Introduction

Infants start life by being broadly attracted to most language signals (Colombo and Bundy, 1981; Vouloumanos and Werker, 2004), they soon undergo perceptual narrowing to the properties of their native language by their first birthday, and their perception of language continues to be honed by their home language experience (Werker and Tees, 1984; Kuhl, 2004; Werker and Curtin, 2005; Kuhl and Rivera-Gaxiola, 2008).^1^ The attraction to language signals and the subsequent tuning to language-specific linguistic properties is observed not only in spoken languages but in signed languages as well (Baker et al., 2006; Krentz and Corina, 2008; Palmer et al., 2012). It is widely believed that this process is enabled by infants’ selective attention to distinctive communicative signals and statistical patterns in their environments (Saffran et al., 1996). Understanding language development is well studied from an auditory-speech perspective (e.g., Jusczyk, 1997, 2016), but language is rarely singularly heard without looking at a speaker’s face, talking mouth, and gesticulating body. Face-to-face communication is inherently multimodal. Infants and children need to learn what parts of their acoustic and visual worlds are linguistically relevant; this is a puzzle given that humans engage in constant vocal and body movements, some of which are gestures or signs used to communicate. Early perceptual attunement from this multimodal perspective is not well understood. In Experiment 1, we examined whether infants show selective visual attention (by means of differential gaze patterns) to different classes of body actions at two ages, 6 and 11 months and, in Experiment 2, we examined whether this sensitivity is shaped by the modality of language experience in young children between 2 and 8 years of age.

Findings from developmental studies indicate that infants can distinguish and derive meaning from classes of human body actions. Young infants aged from 5 to 9 months are sensitive to the goal-directed nature of manual reaching and grasping (Woodward, 1998; Woodward et al., 2001; Behne et al., 2005; Reid et al., 2007; Daum et al., 2009). They also have expectations about how the body and arms are supposed to move (Komori, 2006; Christie and Slaughter, 2010; Morita et al., 2012; Hannon et al., 2017). By 10–12 months, they can make sense of the intent of novel body action behaviors from video (Meltzoff, 1995, 1999; Wellman and Phillips, 2001; Csibra et al., 2003). They also make use of gaze direction, gestures, body posture, and emotional expressions to guide such intentional inferences (Tomasello, 1999; Baldwin, 2016). Although infants acquire the sense of body action perception in the first year of life, other recent studies suggest that infants struggle to make the leap to understand body actions as symbolic *representations* (Novack et al., 2018). This ability may require mastering certain language milestones and/or acquiring knowledge about how objects are used before understanding body actions as communicative gestures (Novack and Goldin-Meadow, 2017). For instance, toddlers around age 1–2 years imitate the goals of other person’s actions and visually anticipate other’s future actions (Hamlin et al., 2008; Cannon and Woodward, 2012), *but* when shown an instrumental body action (such as hammering with no object), and asked to pick one of the two objects, they pick the correct instrument no greater than chance (Namy, 2008; Novack et al., 2018). This ability to connect a symbolic gesture and its referent is not reliably in place until about 2 and 5 years of age (Namy, 2008; Goodrich and Hudson Kam, 2009; Dimitrova et al., 2017).

In other recent studies, infants’ attention to talkers’ faces is intricately linked to language developmental milestones and is modulated by language experience, such as bilingualism. Between 6 and 8 months, the infants attend to the talker’s mouth; at 12 months, attention shifts to the eyes *unless* they view a silent talker of an unfamiliar language; then they continue to attend to the mouth (Lewkowicz and Hansen-Tift, 2012; Tenenbaum et al., 2013). The explanation offered for this shift is that infants look for articulatory cues (i.e., the mouth) at a time when they have not yet mastered speech production; after this developmental stage, they shift to focus on social body cues, i.e., the eyes (Lewkowicz and Hansen-Tift, 2012; Rutherford et al., 2015). It is to be noted that these studies typically show the head of a talker, without a stationary or gesticulating body. Nonetheless, these findings are critical because visual attention to the face in the first year of life has emerged as a meaningful predictor of later social and language developmental outcomes in toddlers and preschoolers (Morales et al., 1998; Brooks and Meltzoff, 2008; Young et al., 2009; Chawarska et al., 2013; Tenenbaum et al., 2014; Peltola et al., 2018; Morin-Lessard et al., 2019).

Together, this body of research on the development of visual attention patterns in infants supports a notion of developmental shifts in the sensitivity to, and understanding of, communicative human actions conveyed through speakers’ bodies and faces. That is, once infants gain an understanding of the biomechanical constraints and basic functional properties of human actions, they shift to understand body actions as carriers of causal intention and meaning (Meltzoff, 1995, 1999; Wellman and Phillips, 2001). Infants’ gaze patterns to faces demonstrate their understanding of the relationships between articulatory facial movements and speech while later index their awareness of a social-dyadic communication system in which the interlocutors’ eyes hold informative clues. However, what happens when the primary mode of articulation is not the mouth but the *hands*, and how does experience with a visual language modality influence early understanding of body actions? We know little about the developmental changes that arise when human body actions are systematized as linguistic communicative signals, as in the case of naturally occurring signed languages. Contrasting signed-manual or spoken-oral modalities of language transmission can provide a critical test of current cognitive developmental theories. Two fundamental questions are addressed in the present study: *First*, do infants’ visual attention reveal sensitivity to different classes of human actions (e.g., visual-manual language as compared to self-directed body actions and symbolic pantomime)? *Second*, does language experience as spoken or signed influence visual attention to body actions in young children?

Signed languages are structurally complex, naturally evolving communicative systems used by deaf people and acquired by hearing children of deaf adults (CODAs) as a first language with the same timeline as children learning a spoken language (Petitto and Marentette, 1991; Lillo-Martin, 1999; Rinaldi et al., 2014; Newport and Meier, 2017). Within the field, there is very good consensus that signed languages display core linguistic properties that are characteristic of those identified in spoken languages (Stokoe, 1960; Klima and Bellugi, 1979; Sandler and Lillo-Martin, 2006; and refer to Pfau et al., 2012 for a review). Moreover, there is substantial evidence that the cognitive processes involved in signed and spoken language are qualitatively similar, such as the mapping between perceptual forms (either visual or auditory) and stored lexical representations, the activation of phonological forms and lexical-semantic meaning, and the involvement of attention and memory processes engaged during the parsing and comprehension of linguistic forms. In addition, there is well-established evidence for commonalities in the core cortical and subcortical brain systems that mediate spoken and signed languages (Emmorey, 2001; Corina and Knapp, 2006; Corina and Blau, 2016; Corina and Lawyer, 2019). One difference from spoken language is the greater prevalence of signed lexical items whose forms are physically motivated through body actions, where the articulation of the form carries transparency about the form’s meaning [e.g., DRINK in American Sign Language (ASL) is similar to how most would communicate the action of drinking through gesture; Ortega, 2017]. Indeed, there is convincing evidence that lexical signs evolved from earlier forms of symbolic manual gestures (Frishberg, 1975; Kegl et al., 1999; Morford and Kegl, 2000; Armstrong and Wilcox, 2003; Goldin-Meadow, 2005; Sandler et al., 2005; Senghas, 2005; Goldin-Meadow and Brentari, 2017). Although signs may have a gestural origin, they differ in systematic ways from pantomimic gestures. First, pantomimic body actions are holistic, with meaning derived from the whole, not parts (McNeill, 1992; Sandler and Lillo-Martin, 2006). They are less conventionalized and more idiosyncratic across individual productions (Wilcox and Occhino, 2016; Lepic and Occhino, 2018). In contrast, lexical signs are conventionalized forms with clear sub-lexical structure built from constrained (and language-specific) inventories of handshapes, orientations, places of articulation on the body, and movement trajectories (reviewed in Wilbur, 1979; Nespor and Sandler, 1999; Brentari et al., 2018). Differences in the features of any of these phonological units result in a different meaning for the sign providing evidence for the duality of patterning seen in the spoken language (Stokoe et al., 1976; MacSweeney et al., 2004). In sum, there are both similarities and differences between gestural body actions and lexical signs that might shape how infants and children perceive and learn them.

Prior studies have revealed that typically hearing non-sign-exposed 6-month-olds are sensitive to visual signed languages. For example, they show preferences for ASL over pantomimed actions (Krentz and Corina, 2008), a preference that is not observed in 10-month-olds. In addition, there is growing evidence for the perceptual narrowing of sensitivity to distinctive components of signed languages. Indeed, sign-naïve infants can categorically perceive a continuum of open-closed handshapes (Baker et al., 2006; Palmer et al., 2012). Infants also look longer at well-formed over ill-formed lexicalized fingerspelling (Stone et al., 2018). Six-month-olds can perceive syllabic reduplication common to linguistic signs, and their neural response differs from visual controls (Berent et al., 2021). Nine-month-old infants are sensitive to intonational phrase boundaries in child-directed-signing (Brentari et al., 2011). These sensitivities have been found to wane by 12 months of age in hearing infants not exposed to sign language (Baker et al., 2006; Krentz and Corina, 2008; Palmer et al., 2012; Stone et al., 2018, but cf. Brentari et al., 2011). While these studies demonstrate that sign-naïve infants show particular preferences for linguistic manual movements, we do not yet know how infants and children extract information from these body action displays to form these biases or whether the information they seek changes over time. In the present study, we use eye tracking methodology to address this gap.

In Experiment 1, we compared gaze patterns in hearing sign-naïve 6-month and 11-month-olds to assess whether they have selective attentional biases for different body action types. Specifically, we contrasted overt visual attention for linguistic body actions (series of lexical ASL signs produced without mouthing or facial expressions), intransitive self-directed body actions (“grooming,” such as scratching face, brushing shoulder, and smoothing hair), and object-directed pantomime body action (“mimes,” such as catching a ball, turning pages of a newspaper, and cracking an egg) created by a native signer. The inclusion of two types of non-linguistic actions (self-grooming and pantomimic) were included to examine whether the symbolic content of the actions might drive changes in eye-gaze behavior. While pantomimes are symbolic, the self-grooming actions lack this quality. The extent to which the participant groups differ in their visual attention across these body action types provides evidence that they are able to differentiate them. Specifically, if body action perception follows evidence of attunement (discussed above), then 6-month-olds, but not 11-month-olds, should show different gaze patterns for the body action types. Moreover, we reasoned the two regions, the face and the articulatory space where the hands produce language, might compete for infants’ attention. On the one hand, infants might have a strong attentional bias for a signer’s face because infants are known to be highly attracted to faces that provide emotional-social cues (Frank et al., 2009, 2014; Reynolds and Roth, 2018). Alternatively, infants might show a strong attentional bias to look at the articulatory space (in front of the torso) where the hands primarily fall.^2^ This is expected because infants do have an attraction to look at perceptually salient moving objects over stationary ones (Slater et al., 1990; Arterberry and Bornstein, 2002). Also, as infants age, they demonstrate increasing interest in looking at hands and anticipate the motion of hands when agents perform actions on objects (Aslin, 2009; Slaughter and Heron-Delaney, 2011; Frank et al., 2012; Reddy et al., 2013).

In Experiment 2, we addressed our second question about whether linguistic experience influences visual attention patterns for different classes of human body action by contrasting native-signing CODAs vs. non-sign-exposed hearing children. As described above, native signers are exposed from birth to a formal visual-manual language that serves as their primary means of communication at home. They also might have extensive experience with pantomimic and gestural communication (Emmorey, 1999). As such, we hypothesized that experience with a visual language may shift visual attention patterns of native signers, making them different from non-signing children. Specifically, group differences would reflect CODAs’ unique social and language knowledge, while non-signing children would be driven by perceptually salient attributes in the stimuli. This is the first study to address this topic in children. All methods were identical for both Experiment 1 (infants) and Experiment 2 (children).

## Experiment 1: Method

### Participants

A total of 46 hearing infants between 5 and 14 months of age were tested. Three participants did not complete testing, 2 were excluded because of poor calibration, and 2 did complete the testing, but were removed for insufficient data. All the remaining 39 infants included in the analysis completed the entire experiment (refer to Table 1). Two groups were tested, 22 6-month-olds (8 males/12 females; mean age = 6.04 mos) and 17 11-month-olds (9 males/8 females; mean age = 10.85 mos). All infants were from monolingual English-speaking homes, and, based on our selection criteria, had typical hearing and no sign language exposure. Race was reported as 67% White, 13% Hispanic, 8% Black, 8% Asian, and 5% mixed.

**TABLE 1:** Demographics of study participants.

Age group	*N*	Male/female	Mean age in months (SE)	Median	Range
Six-month-olds	22	10/12	6.04 (0.12)	6.05	5.0–7.0
Eleven-month-olds	17	9/8	10.85 (0.33)	10.50	7.8–14.0

**Language group**	***N***	**Male/female**	**Mean age in years (SE)**	**Median**	**Range**

Non-signing children	18	8/10	4.77 (0.37)	4.67	2.90–8.02
Native signing children	13	7/6	5.70 (0.68)	5.93	2.08–8.32

Age for infants presented in months and for children in years.

All participants were reported to be healthy and free from neurological impairments or other major disabilities. The Institutional Review Board at UCSD approved the experimental protocol, and written informed consent was obtained from the parents when they arrived at the lab. Testing was completed within a 30-minute visit to the lab before the COVID-19 Pandemic.

### Apparatus

Visual stimuli were presented on a Hewlett-Packard p1230 monitor (1440 × 1080 pixels; 75 Hz) controlled by a Dell Precision T5500 Workstation computer, using Tobii Studio 3.4.2 software. A Tobii 120X eye tracker is a free-standing device positioned in front of the participant, just under the monitor, and was used to track the participant’s near-infrared reflectance of both eyes, with an average gaze position accuracy of 0.57° of visual angle. The tracker provided *x*-*y* coordinates for each eye that corresponded to the observer’s gaze point on the monitor, during stimulus presentation, recorded at a sampling rate of 120 hertz. From these data, we averaged across the eyes to provide binocular eye gaze position in *x*-*y* space, every 8.33 ms.

### Materials

The stimuli consisted of alternating video sequences of three body action types: body grooming (e.g., rubbing hands or fixing hair), pantomimed actions (e.g., clicking a mouse or picking an apple from a tree), and ASL signs (e.g., HOT, FREE, and ASK), produced as citation forms by the same female native signer (refer to Supplementary material for a list of all 56 items). The signing model was given an English glossary of each word or description of the action and made her own natural articulation for each. The signer was instructed to produce all actions and signs with a neutral facial expression. For each token, the signer started with her hands folded in front of her lower torso, produced the token, returned her hands to the same position, paused for 1s, and then produced the next item.

We balanced the number of sign and body action tokens that were executed with one or two hands, had a clear handshape change, and had clear path movements. For each of the three body action types, exactly 50% of tokens had handshape change, and 50% did not. The number of sign and body action tokens with a clear movement path vs. no movement path was equivalent.^3^ The number of tokens with two articulating hands were 71, 71, and 57% for Signs, Mimes and Grooming, respectively, with the rest one-handed. The total video duration of each of the Sign, Mime, and Grooming conditions were 39.14, 44.40, and 58.45 s, respectively.

Viewed from a distance of 65 cm, the height of the signing model was 18.3°, shoulder width was 7.8° of visual angle, and the distance between the center of her eyes was 2°. Videos of the signer were presented upon a full-monitor screen, 1,440 × 1,080 pixels, upon a white background.

### Procedure

The infant sat on a booster seat on the parent’s lap. The parent wore glasses with opaque filters and was discouraged from interacting with their child unless necessary. The experimenter sat behind a curtain, unseen by the participant.

**FIGURE 1:**
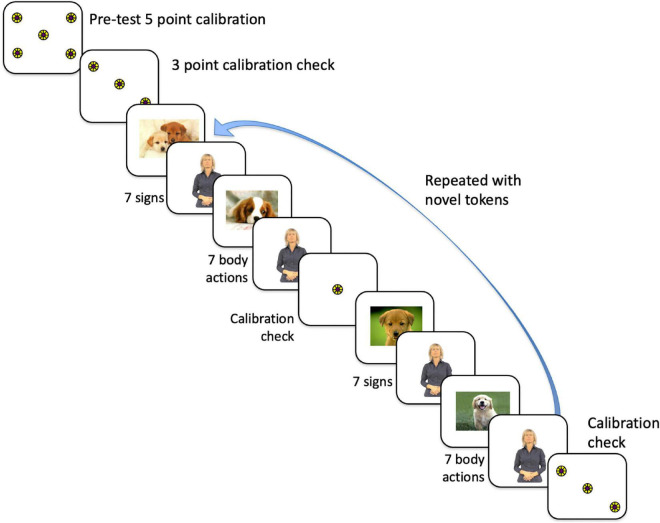
Order of presentation of stimuli to participants, which first commenced with a 5-point calibration routine, then a 3-point calibration check. We proceeded with the experiment only if that calibration was within the tolerance limits (with gaze falling on each circle). The experimental conditions consisted of alternating trials of 7 signs and trials of 7 body actions, either grooming or pantomimes, with each trial presented twice. Participants never saw the same token twice. For the data analysis, the trials were collapsed to eliminate order effects.

Participants were calibrated using a 5-point calibration procedure, using a small spinning pinwheel circle presented for 1–3 s in each of 5 locations (see Figure 1). Once the participant was successfully calibrated, we recorded gaze data for these circle targets, which was used off-line to verify calibration accuracy. Visual inspection showed no discernable drifts or significant changes in calibration for any participant.

Refer to Figure 1 for the timeline of the experiment. The total experiment lasted approximately 7 min. The 8 trials (each with 7 tokens) were interspersed with a still picture of a dog in the center of the monitor. When the participant’s eye gaze was centered on the dog, the experimenter initiated the test trial. Participants saw each Body Action Type with the order of condition counterbalanced. Counterbalanced group assignment alternated for each consecutive subject. Data analysis was done with counterbalanced groups collapsed in an effort to control for order effects.

### Data analysis

#### Raw gaze data

Raw eye gaze data in *x*-*y* form, indicating horizontal (*x*) and vertical (*y*) positions in 2-D space, were obtained for each eye, and averaged across both eyes. The four trials for each condition were combined for all analyses to protect against order effects. To examine whether the groups demonstrated different *overall looking times* for the various body action types, an ANOVA was conducted with between-subjects factor Age Group (6-, 11-months) and repeated-subjects factor Body Action Type (Grooming, Mimes, and Signs).

#### Area of interest analyses

**FIGURE 2:**
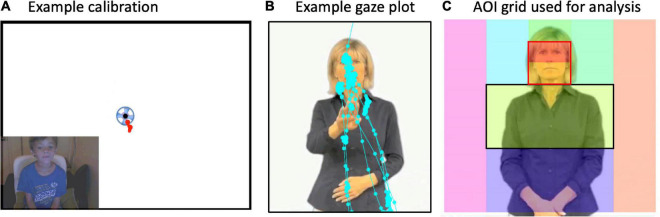
**(A)** Example calibration from a child. If the gaze “hit” the spinning circle at each beginning, middle, and at the end of the experiment, then data were included in analyses. **(B)** Example “raw” gaze plot showing fixation points from one trial and one participant. **(C)** The signer was superimposed with an Areas of Interest (AOI) grid. Gaze points in each box were summed to equal the total time spent gazing at each AOI box. Then, for main analyses, percent looking in each AOI box was computed as total looking time spent in the AOI divided by the total amount of time spent looking at the whole image (i.e., all boxes). Gaze data primarily hit the midline column of AOIs and rarely off the signer’s body. Main analyses were conducted on face preference index (FPI) values. FPIs were calculated for each participant as the Face AOI (outlined in *red*) divided by the Face and “Torso” AOIs (outlined in *black*).

**FIGURE 3:**
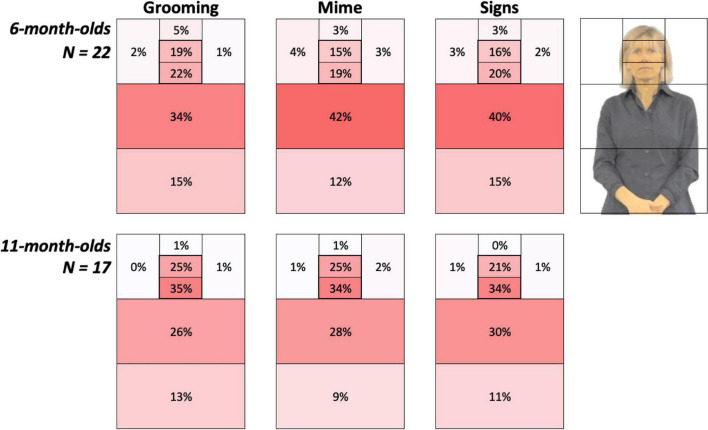
Heat Grids for each age group and Body Action type conditions. These results show the average percent looking time for each AOI, separately for 6-month-olds (*top*) and 11-month-olds (*bottom*). Color scaling per cell refers to a gradient from the highest (red) to the lowest (white) percent looking values. The outline in the upper right corner represents the AOI locations on the signer. Each grid, including the Left and Right side of AOIs, sums to 100%. As discussed in the Results, 6-month-olds were more drawn to the articulatory space while 11-month-old infants spent 19–25% more time attending to the face.

**FIGURE 4:**
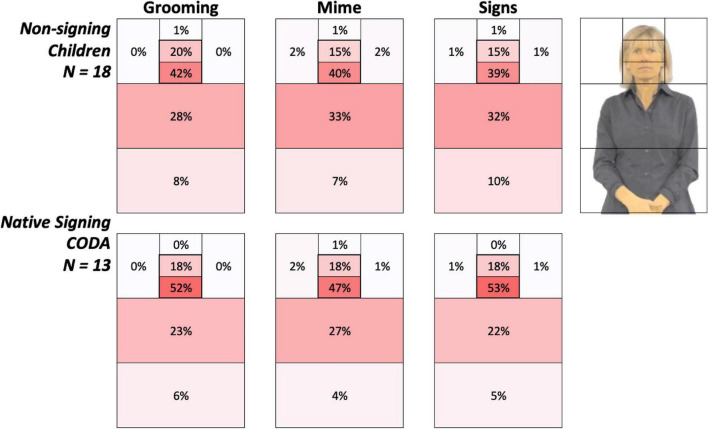
Heat grids for each language group and body action type conditions. These results show the average percent looking time for each AOI for non-signing (*top*) and native signing children (*bottom*). Color scaling per cell refers to a gradient from the highest (red) to the lowest (white) percent looking values. The most notable overall difference between groups was the greater attention to the face in native signing children, especially for signs, than the non-signing children. Conversely, this also reflects non-signing children’s higher percentage looking at the articulatory space.

To examine where participants look, we created a grid of Areas of Interest (AOI) boxes superimposed upon the image of the signer (Figure 2). AOIs were drawn using Tobii Studio Pro software. The grid was dynamically “locked” onto the signer’s body such that when she moved (albeit slightly), the grid moved with her. In this way, the boxes always were linked anatomically to a region of her body (i.e., the “mouth” box is always centered to her mouth). Gaze samples (e.g., eye position every 8.33 ms) were summed as hits for each AOI box. For purposes of illustration, we present summary gaze patterns for all AOIs in the entire grid in Figures 3 and 4. Most of the gaze data fell on the signer’s body, with very few gaze points outside the signer’s body region. As such, we concentrated our analyses on the face (later divided into mouth and eyes) and the torso, which is the primary “articulator” space in front of the singer where the articulators fall the majority of the time.^4^

#### Face preference index values

We explored statistical differences in where participants look at the signer by computing, for each participant, a *face preference index* (FPI) with percent looking time values, as (Face – Torso)/(Face + Torso); refer to Figure 2. This was motivated by a practical desire to reduce the number of comparisons and to test our primary hypotheses about relative visual attention (by means of gaze) to the *face vs. articulatory space* (torso). The face and the moving hands are both highly salient cues that may compete for participants’ attention. The hands, which we refer to globally as the “articulators,” primarily fall in the torso region, commonly called “signing space.” Positive values reflect greater looking at the face than the region below the face. With these FPI values, we could test the prediction that participants might be primarily drawn to either the signer’s face or the signer’s moving hands (or both equally so). Further, we can test predictions about whether the participants look at different parts of the signer for the three different body action types. If they do, this is evidence that they are sensitive to the differences between these stimulus types.

Face preference index data were analyzed first with a mixed 2 × 3 ANOVA, with between-subjects factor Age Group (6- vs. 11-month-olds) × repeated-subjects factor Body Action Type (Grooming, Mimes, and Signs). Planned comparisons for visual attention to Grooming, Mimes, and Signs were conducted using a one-way ANOVA with each participant group.

Levene’s tests of homogeneity of variances were found to be equal, *p* > 0.24. We observed no visible order effects and confirmed no significant differences between Video Groups 1 and 2 (used to counterbalance the presentation of tokens and condition order), nor were there interactions of any factors with Video Group.

## Experiment 1: Results

### Overall visual attention to body action types

In terms of the total number of gaze samples provided, 6- and 11-month-old infant groups provided, on average, 75.04 (SE = 5.85) s and 86.16 (SE = 4.42) s of the total gaze data. There was no difference between the two age groups, *F*(1,37) = 2.08, *p* = 0.16, η^2^ = 0.05.

Infants’ percent looking averages for each condition are presented in Table 2. First, we checked whether the body action types varied in capturing the *overall interest*, irrespective of where one looks (our main interest). To this end, we conducted a 2 × 3 ANOVA with between-subjects factor Age Group (6, 11-month-olds) and repeated-subjects factor Body Action Type (Grooming, Mime, and Signs) with total percent looking at each stimulus condition, collapsed across all trials and AOIs. There was no main effect of Age Group, *F*(1,37) = 2.04; *p* = 0.16; η^2^ = 0.05 or interaction with this factor, *F*(2,74) = 0.36; *p* = 0.70, η^2^ = 0.01. This means that the two age groups did not differ in the overall attentiveness, cooperation, or interest across the three body action types.

**TABLE 2:** Percent gaze recorded for each stimulus condition, normalized by video duration.

Participant group	Grooming	Mime	ASL signs
6-month-olds (*N* = 22)	45.91% (5.00)	56.64% (4.34)	53.80% (3.32)
11-month-olds (*N* = 17)	56.69% (5.68)	62.99% (4.95)	60.08% (3.77)
Non-signing children (*N* = 18)	76.52% (6.78)	73.29% (4.08)	68.27% (4.78)
Native signing children (*N* = 13)	72.34% (6.12)	65.05% (9.67)	73.07% (7.62)

Averages and standard errors of the mean are presented.

### Visual attention to face vs. articulatory space

Figure 3 provides color-coded illustrations of the average percent looking times for each AOI, with each participant’s AOI grid summing to 100%. Darker regions indicate AOIs that contained the greatest number of gaze points and attracted the most attention. These figures show 6-month-olds were more drawn to the articulatory space, while 11-month-old infants spent more time attending to the face.

In the main analysis, we asked where participants spend their time looking, which we divided into two central regions, the *Face* and *Torso* regions. As discussed earlier, we reasoned that these two regions might compete for infants’ attention, and this might depend on body action types. We statistically analyzed the distribution of attention and whether this depended on body action types using FPI values. Participants might have a positive FPI value because infants are known to be highly attracted to faces, especially of talkers and signers. Conversely, if infants are drawn to attend to perceptually salient parts of the image, such as the moving hands, they would show a negative FPI value.^5^

**FIGURE 5:**
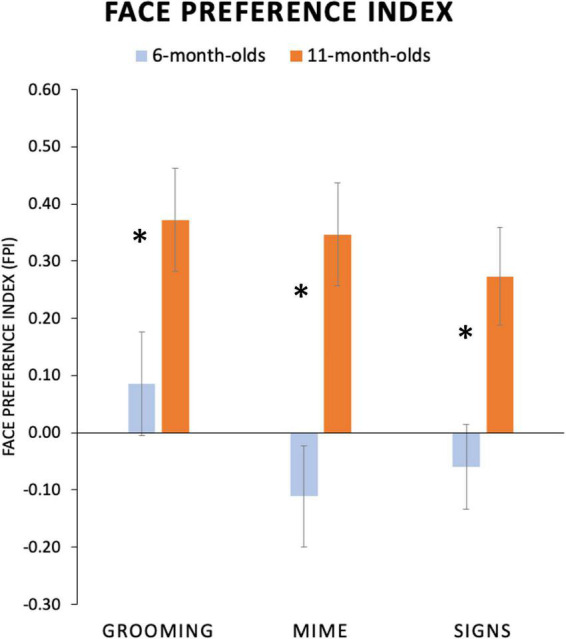
Average face preference index (FPI) values for each age group and condition. FPI values are plotted on the *y-*axis, with positive values indicating greater attention devoted to the face than the torso area and negative values indicating the opposite preference. Six-month-old infants attended to the face for Grooming body actions and to the torso region for both Mimes and Signs. Eleven-month-old infants showed an evenly high face preferences for all conditions. (Standard error bars plotted*, *p* < 0.05).

We conducted an ANOVA on FPI values from each participant with two factors, between-subjects factor Age Group (6-, 11-month-olds) and repeated-subjects factor Body Action Type (Grooming, Mimes, and Signs). Significant main effects were found for both Age Group, *F*(1,36) = 9.01; *p* = 0.005; η^2^ = 0.20, and Body Action Type, *F*(2,72) = 5.44; *p* = 0.006; η^2^ = 0.13. The Age Group and Body Action Type interaction showed a non-significant trend, *F*(2,72) = 2.23; *p* = 0.11; η^2^ = 0.06. As shown in Figure 5, this lack of interaction was driven by *11-month-olds* showing uniformly highly positive FPI values (i.e., a robust face attentional bias) for all body action types. Refer to Table 3 for mean FPI values.

**TABLE 3:** Mean face preference index (FPI) values for each group and condition.

Participant group	Grooming	Mime	ASL signs
6-month-olds (*N* = 22)	0.086 (0.09)	−0.108 (0.09)	−0.062 (0.08)
11-month-olds (*N* = 17)	0.372 (0.10)	0.347 (0.10)	0.277 (0.09)
Non-signing children (*N* = 18)	0.379 (0.07)	0.22 (0.08)	0.241 (0.08)
Native signing children (*N* = 13)	0.478 (0.08)	0.40 (0.09)	0.513 (0.09)

Standard errors of the mean are presented.

Although the overall interaction did not reach significance, based upon our hypotheses about attunement discussed above, we explored this trend by conducting a one-way ANOVA with repeated-subjects factor Body Action Type (Grooming, Mimes, and Signs) separately for each age group as a test of the specific prediction that younger infants would have different visual attention patterns for body action types. Indeed, as predicted, the *6-month-olds* revealed a significant main effect of Body Action Type, *F*(2,40) = 6.60; *p* = 0.003; η^2^ = 0.25, while *11-month-olds* did not, *F*(2,32) = 1.42; *p* = 0.26, η^2^ = 0.08. Specifically, younger infants showed a significantly higher face preference for *Grooming* compared to *Mimes* (Mean Difference = 0.20, *p* = 0.004, 95% CI [0.07, 0.32]) and *Signs* (Mean Difference = 0.15, *p* = 0.02, 95% CI [0.03, 0.27]). Mean FPIs for Mimes vs. Signs were not significantly different (Mean Difference = −0.05, *p* = 0.38; CI [-0.15, 0.06]). None of these contrasts in the *11-month-olds* were significant, with all *p*-values > 0.20.

### Attention to the eyes, mouth, and articulatory space

We followed the main analysis with an exploration of visual attention (in terms of percent looking) to the eyes vs. mouth (which together make up the face in the FPI analyses) and whether this pattern was related to age. Specifically, we examined the correlation of age with percent looking time for the key AOIs analyzed above, the eyes, mouth, and torso.

In all infants tested, who ranged in age from 5.0 to 14.0 months, attention to the *mouth* increased with age (*r* = 0.49; *p* = 0.001), matched with a corresponding decrease in attention to the *torso* (*r* = −0.33; *p* = 0.04), while looking at the *eyes* remained stable with age (*r* = 0.15; *p* = 0.34). Refer to the mean values in Table 4.

**TABLE 4:** Mean percentage looking values for each group, collapsed across body action type.

Participant group	Eyes	Mouth	Torso (Articulatory space)
6-month-olds (*N* = 22)	16.20% (2.81)	20.49% (2.30)	38.95% (3.31)
11-month-olds (*N* = 17)	23.74% (5.07)	34.21% (3.53)	27.91% (3.32)
Non-signing Children (*N* = 18)	16.97% (2.94)	40.29% (4.05)	30.75% (2.97)
Native Signing Children (*N* = 13)	17.71% (2.85)	51.14% (2.71)	24.19% (2.59)

Standard errors of the mean are presented.

## Experiment 1: Discussion

Results showed that hearing sign-naïve 6-month-olds were drawn more to the articulatory space, while 11-month-old infants spent more time attending to the face. Moreover, 6-month-old infants showed differential visual attention for Grooming compared to Mimes and Signs, while 11-month-olds showed uniformly robust face attentional bias for all body action types. This pattern suggests an early perceptual sensitivity to classes of body actions that wanes around one year of age in the absence of signed language exposure, also recently reported for the perception of handshapes (Baker et al., 2006; Palmer et al., 2012; Stone et al., 2018). Exploratory analyses indicated that across the ages of 5–14 months, attention to the mouth increased, mirrored with a decrease in attention to the articulatory space where the hands primarily fall, while looking at the eyes remained stable with age.

We now turn to Experiment 2 to address the question about whether linguistic experience influences gaze preference for different classes of human body action by contrasting native-signing CODAs vs. non-sign-exposed hearing children. Native signers are exposed from birth to a formal visual-manual language that serves as their primary means of communication at home. We hypothesized that experience with a visual language may shift visual attention patterns for native signers, making them different from non-signing children. All methods were identical for both Experiment 1 (infants) and Experiment 2 (children).

## Experiment 2

All stimuli and procedures are identical to Experiment 1.

### Participants

A total of 35 hearing children were tested. Two participants’ data failed to be recorded due to experimenter error, and an additional 2 were removed for poor calibration. All the remaining 31 children between 2 and 8 years of age (mean age of 5.16 years) included in the analysis completed the entire experiment (refer to Table 1). One group of 18 children (8 males/10 females) were monolingual English speaking at home and, based on our selection criteria, had no sign language exposure. The other group consisted of 13 “CODAs”; (7 males/6 females) whose deaf parents’ primary language was ASL. CODAs are typically considered *native signers*. Parents self-reported that they used ASL as their primary language and used it at least 80% of the time. Prior to testing, all deaf parents completed a self-rated proficiency test, taken from Bosworth et al. (2020). All deaf parents gave themselves the maximum rating of 5. We did not assess the language fluency in children. All participants were reported to be healthy and free from neurological impairments or other major disabilities.

The mean ages of the non-signing and native signing groups were 4.77 and 5.70 years, respectively, and did not differ significantly in age, *F*(1,29) = 2.020; *p* = 0.166; η^2^ = 0.065. Race was reported as 44% White, 15% Hispanic, 18% Black, 0% Asian, 3% mixed, and 20% not reported.

The children completed the Matrices subtest of the Kaufman Brief Intelligence Test, 2nd Edition (K-BIT2; Kaufman and Kaufman, 2004), which is an index of non-verbal intelligence. The two groups of non-signing and native signing children did not differ significantly in this test, *p* > 0.20.

The Institutional Review Board at UCSD approved the experimental protocol, and written informed consent was obtained from the parents when they arrived at the lab. Testing was completed within a 30-min visit to the lab before the COVID-19 Pandemic.

### Procedure

The children sat alone on a chair. The tester used spoken English with non-signing children and both English and ASL with the native signing children. Children were instructed to simply watch the video which they might find enjoyable. All other procedures were identical to Experiment 1.

### Data analysis

Raw eye gaze data were processed as described above in Experiment 1. An ANOVA was conducted with between-subjects factor Language Group (Non-signing and Signing) and repeated-subjects factor Body Action Type (Grooming, Mimes, and Signs). To examine where participants look, we created a grid of AOI boxes superimposed upon the image of the signer (refer to Figure 2). For purposes of illustration, we present summary gaze patterns for all AOIs in the entire grid in Figure 4. Most of the gaze data fell on the signer’s body, with very few gaze points outside the signer’s body region. As with Experiment 1, we concentrated our analyses on the face (later divided into mouth and eyes) and the torso, which is the primary “articulator” space in front of the singer where the articulators fall the majority of the time.

*FPI Values.* We explored statistical differences in where participants look on the signer by computing, for each participant, an FPI with percent looking time values, as (Face – Torso)/(Face + Torso). With these FPI values, we tested the prediction that language groups differ in where their attention is drawn, either the signer’s face or the signer’s moving hands (or both equally so). Further, we tested predictions about whether the participants look at different parts of the signer for the three different body action types.

Face preference index data were analyzed first with a mixed 2 × 3 ANOVA, with between-subjects factor Language Group (Non-signing, Signing) × repeated-subjects factor Body Action Type (Grooming, Mimes, and Signs). Planned comparisons for visual attention to Grooming, Mimes, and Signs were conducted using a one-way ANOVA with each participant group.

Levene’s tests of homogeneity of variances were found to be equal, *p* > 0.24. We observed no visible order effects and confirmed no significant differences between Video Groups 1 and 2 (used to counterbalance the presentation of tokens and condition order), nor were there interactions of any factors with the Video Group.

## Experiment 2: Results

### Overall visual attention to body action types

Non-signing and native signing children provided, on average, 104.01 (SE = 6.20) s and 101.76 (SE = 10.77) s of total gaze data, respectively. There was no difference between the two Language Groups in the total amount of gaze data provided, *F*(1,29) = 0.04; *p* = 0.85; η^2^ = 0.001.

Using total percentage looking at the stimuli, ANOVA results showed no main effect of Language Group, *F*(1,29) = 0.097; *p* = 0.76, η^2^ = 0.76 or main effect of Body Action Type, *F*(2,58) = 1.07; *p* = 0.35, η^2^ = 0.036, and no higher order interaction, *F*(2,58) = 1.63; *p* = 0.21, η^2^ = 0.05. As such, there were no differences in the overall interest for the stimuli between the two participant groups or for the body action types (refer to Table 2). Both groups were equally interested and cooperative in viewing the stimuli. Even if they do not know ASL, they seemed to have high interest in watching it.

### Face preference index results

Figure 4 provides color-coded illustrations of the average percent looking times for each AOI, with each participant’s AOI grid summing to 100%. Darker regions indicate AOIs that contained the greatest number of gaze points and attracted the most attention. The most notable overall difference between the groups was greater attention to the face in native signing children, especially for signs, than non-signing children. Conversely, this also reflects non-signing children’s higher percentage looking at the articulatory space.

To examine the effects of ASL exposure on gaze patterns, we conducted a repeated measures ANOVA with between-group factor Language Group (Non-signing, Signing) and within-subject factor Body Action type (Grooming, Mimes, and Signs) using FPI values as the dependent measure. We first included age as a covariate, as age was neither significant, *F*(1,26) = 0.798, *p* = 0.38, η^2^ = 0.03, nor did it interact with any factors, we dropped this factor. A significant main effect of Body Action Type, *F*(2,54) = 3.72; *p* = 0.03; η^2^ = 0.12, and a marginal trend for the factor Language Group, *F*(1,27) = 3.12; *p* = 0.08; η^2^ = 0.10, were found. There was no interaction between Body Action Type and Language Group, *F*(2,54) = 1.96; *p* = 0.15; η^2^ = 0.07. Refer to Table 3 for mean FPI values.

**FIGURE 6:**
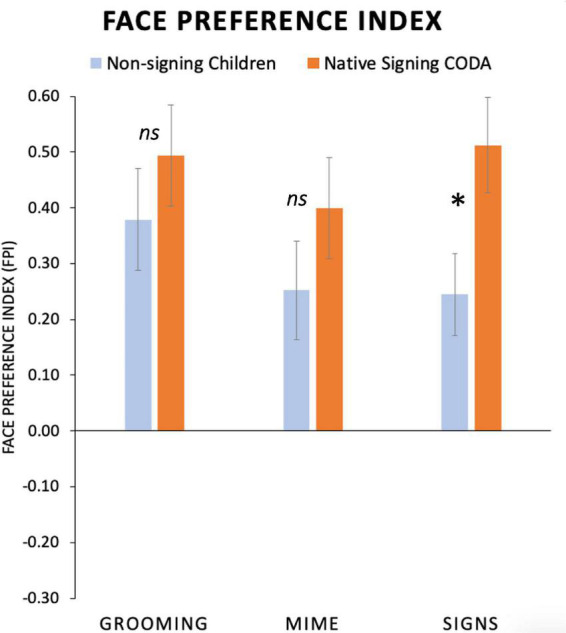
Average face preference index (FPI) values for non-signing and signing young children at the mean age of 5 years. Positive FPI values indicate greater attention devoted to the face than the torso area and negative values indicate the opposite preference. All participants had a high positive FPI. No group differences were seen for Grooming or Mime actions. For Signs, native signing children had a significantly greater FPI than the non-signing children. (Standard error bars plotted*, *p* < 0.05). ns, not significant.

Average FPI values are presented in Figure 6, separately for each participant group and for the three Body Action Type conditions. As shown in Figure 6, all child participants had positive FPI (i.e., a face preference) values for all body action types. We predicted that the native signing children would have a different gaze pattern than the non-signing children, and perhaps this would be different for the body action types. Indeed, native signing children had a significantly higher mean FPI than non-signing children did for *Signs*, (Mean Difference = 0.26, *p* = 0.02; 95% CI [0.04, 0.48]), and there were no group differences for *Grooming*, *p* = 0.36, or *Mimes*, *p* = 0.15.

### Attention to the eyes, mouth, and articulatory space

We followed the main analysis with an exploration of visual attention (in terms of percent looking) to the eyes vs. mouth (which together make up the face in the FPI analyses) and whether this pattern was related to age. Specifically, we examined the correlation of age with percent looking time for the key AOIs analyzed above, the eyes, mouth, and torso.

In the children tested, who ranged from 2 to 8 years of age, there were no significant correlations with age (all *p*-values > 0.20). As shown in Figure 4, native signing children spent much more time attending to the *mouth*, compared to the non-signing children (51.14% vs. 40.29%, *p* = 0.03) and less attention to the *torso* region (24.20 vs. 30.75%, *p* = 0.11), while both groups looked at the *eyes* the same amount of time (17.71 vs. 16.97%, *p* = 0.86). Refer to the mean values in Table 4.

## Experiment 2: Discussion

We predicted that the native signing children would have a different gaze pattern than the non-signing children, and perhaps this would be different for the body action types. Indeed, across all body action types, native signing children had a significantly higher mean face attention than the non-signing children did. This group difference was also significant for signs, while there were no group differences for the two non-linguistic body actions, grooming or mimes. Exploratory analyses indicated that native signing children spent much more time attending to the mouth and less time looking at the articulatory space, compared to the non-signing children, while both groups looked at the eyes in the same amount of time.

## General discussion

The present study tested whether young pre-linguistic infants have differential visual attention patterns for linguistic and non-linguistic body action types and whether this was modulated by age. In older children, we then examined whether a child’s home language as visual-signed vs. spoken-auditory changes their visual attention for these forms. We contrasted gaze patterns for three classes of human actions presented as video sequences of ASL signs, self-oriented manual body actions (grooming, e.g., scratch neck and rub shoulder), and object-oriented pantomimes (mimes, e.g., tie a ribbon and turn a newspaper). We reasoned that if where one looks (i.e., overt visual attention) differs across these body action types, then this provides evidence that the infants and children can perceptually discriminate between them. An important strength of the current study is all stimuli were produced naturally, yet with necessary controls for perceptual matching, such as the similar use of articulatory space, use of two vs. one hand, and without mouthing, facial expression, or narrative prosody. This is important because narrative prosody is perceptually different in many ways from other body actions, which would make it difficult to contrast perception across body action types.

We found that 6-month-old infants showed greater attention to the articulatory space of a signer producing signs and mimes but more face-focused attention for grooming actions. This contrasts with the 11-month-olds who showed a uniformly robust attentional bias for the face, with no difference in the gaze behavior for linguistic vs. non-linguistic body action types. Native signing children exposed to a visual language at home had a significantly greater face attentional bias than non-signing children for ASL signs, but not for grooming and mimes. Together, these findings suggest the following important interpretations: young sign-naïve infants between 5- and 7-months of age can discriminate between visual linguistic and non-linguistic body types. This pattern of body action sensitivity diminishes between 8 and 14 months of age, presumably because they are not exposed to a visual-manual language, suggesting that the well-known attunement phenomenon is modality-general. Results from children between the ages of 2 and 8 years suggest that the modality of language experience in the home alters visual attention for visual-manual linguistic body actions. We address each potential interpretation in turn below.

Our results suggest that young sign-naïve infants, with minimal world experience, can discriminate linguistic signs from self-directed manual grooming body actions. This finding extends the well-known early hypersensitivity to acoustic and visual contrasts for unfamiliar language input and those within their home environment (reviewed in Kuhl, 2004; Vouloumanos and Werker, 2004). For example, young infants prefer native speech over non-speech prosody (Mehler et al., 1988) and signed stimuli over non-sign manual movements(e.g., Krentz and Corina, 2008). Infants at this age are sensitive to well-formed specific handshape and movement paths that adhere to linguistic rules in signed contrasts presented on the hands (Baker et al., 2006; Brentari, 2010; Palmer et al., 2010; Stone et al., 2018; Berent et al., 2021). These findings suggest that early perceptual sensitivity is *amodal*, such that infants are able to pick up on potentially relevant linguistic contrasts in either auditory or visual modalities. This sensitivity is precocious and supports later acquisition of words, concepts, and the relations between them (Yeung and Werker, 2009; Perszyk and Waxman, 2018). This early amodal sensitivity lays the foundation for the identical maturational patterns and timetable of the stages of language learning seen in both speaking and signing children (Bellugi and Klima, 1982; Newport and Meier, 1985; Petitto and Marentette, 1991; Lillo-Martin, 1999; Meier, 2002; Mayberry and Squires, 2006; Pichler et al., 2018).

While infants differentiated signs from grooming, they did not show differential visual attention to signs and mimes (refer to Figure 5). How did our stimuli differ in a way that infants could potentially identify grooming as perceptually distinctive from mimes and signs? First, we have a sense of what is *not* driving this effect from our description of the stimuli (described in Supplementary material). All body action types were closely matched in overall signing space, use of one vs. two hands, and whether the hands changed shape (including opening and closing). Also, all stimuli had no mouthing, body sway, or facial expression, so those attributes are unlikely to be driving infants’ attentional differences in the present study. What differed between grooming vs. signs and mimes is likely the variation and complexity of handshapes. For instance, the mime stimuli employed more handling-like handshapes and more crisp handshapes, while grooming had few handlings and more lax handshapes. Another important difference is the role of the “self,” the grooming actions involve the hands largely directed to the self, intentionally performing an act on or to the body, while the mimes and signs mostly are movements away from the signer and are executed for the sake of perception to “others.” The ASL has many depicted actions that are very “mime-like” (Dudis, 2004), so having an ASL native signer execute the pantomimes might have influenced the execution of these forms to be more “sign-like” or communicative.

Another possible explanation for why infants did not differentiate mimes from signs, but did from grooming, is that perhaps more experience is needed to understand handling objects depicted in mimes.^6^ Although infants acquire body action perception sense in the first year of life, studies suggest that infants do not understand body action as symbolic representation until after the first year of life (Novack et al., 2018). Around 10–12 months, but not before, infants can recognize the intentionality of body action behaviors on video and infer intention from gestures and body posture (Meltzoff, 1995, 1999; Tomasello, 1999; Wellman and Phillips, 2001; Phillips and Wellman, 2005; Baldwin, 2016). This ability to understand *instrumental* object-directed body actions may require mastering certain language milestones and/or acquiring knowledge about how objects are used before understanding body actions as communicative gestures (see Namy, 2008; Novack and Goldin-Meadow, 2017; Novack et al., 2018).

Our results from the older infants showing no differentiation in their visual attention patterns across body action types support the well-documented attunement that starts around eight months of age. This phenomenon has also been observed in an initial global preference for foreign speech that hones into a preference for native language prosody (Mehler et al., 1988; Nazzi et al., 1998, 2000). Our findings also contribute to the recent growing evidence that this phenomenon applies to *visual* modality as well. In these studies, sign-naïve 10- to 12-month-olds did not show a visual sign language preference (Krentz and Corina, 2008) or for either well-formed or ill-formed fingerspelling (Stone et al., 2018) that 6-month-olds did. Also, sensitivity to signed contrasts diminishes by 14 months of age without signed exposure (Baker et al., 2006; Palmer et al., 2012). Together, these findings suggest that young infants are sensitive to visual language, but without sign language exposure, the visual-manual modality is no longer a linguistic domain for them, as their attention, interest, and sensitivity hone to their native spoken language. Although we do not know what would happen with native signing 12-month-olds who hone in preference to their native sign language, recent studies suggest that native signing infants have mature visual attention patterns for social and linguistic signals in place by one year of age (Brooks et al., 2020; Bosworth and Stone, 2021).

Linguistic experience shapes visual attention to body action types, as seen by the present results comparing native signing children raised with ASL with monolingual English-speaking children. As shown in Figure 6, native signing children have significantly higher face-focus than non-signing children for signs, and there were no group differences for grooming or mimes. Examination of Figure 4 shows that both groups look at the eyes for about the same amount of time while signing children spend much more time on the mouth region. Other evidence using displays of silent talking speakers also shows that visual attention to the face is shaped by bilingualism (Weikum et al., 2007; Pons et al., 2015; Mercure et al., 2018, 2019; Birules et al., 2019). The signing children’s high attention to the face is very similar to that seen in native deaf adult signers in a companion study (Bosworth et al., 2020). In that study, adult signers who learned ASL in early childhood had the same robust face-focused attention when watching signed narratives, while adult novice signers’ gaze was variable, especially for low-intelligibility stimuli. The fact that signers rarely foveate to the articulatory space (in front of the torso) means that the details of the hands primarily fall in the peripheral lower vision. This may explain why native signers develop an efficient perceptual “span” that becomes entrained with sign language exposure and leads to heightened visual sensitivity for the articulatory space (Caselli et al., 2022). Indeed, face processing and perception of the inferior visual field have been shown to be enhanced in the deaf and hearing signers compared to non-signers (Bettger et al., 1997; McCullough and Emmorey, 1997; Bosworth and Dobkins, 2002; Stoll et al., 2018, 2019; Stoll and Dye, 2019).

Finally, we also found that sign-naïve 6-month-olds were drawn to look at the articulatory space while 11-month-olds were drawn to the face. In the present study, the body actions were produced in the absence of facial expressions and mouth movements, leaving only phonological information transmitted through hand configurations that change and move in relation to specific locations on the body. We also reported this early attention to manual articulators in sign-naïve infants using signed narratives (Stone and Bosworth, 2019) and ASL fingerspelling (Stone et al., 2018). The present findings of 11-month-olds looking heavily at the face, specifically the mouth, may be related to recent evidence of a developmental shift in infants’ abilities to perceive audiovisual speech and their looking patterns while watching dynamic talker’s faces (Lewkowicz and Ghazanfar, 2006; Pons et al., 2009; Lewkowicz et al., 2010; Grossmann et al., 2012; Lewkowicz, 2014). These studies show that when infants between 10 and 12 months perceive *unfamiliar* non-native speech, they look at the talker’s mouth, but when they look at *familiar* talkers, they focus on the eyes. The explanation is put forth in those studies for this result is that those infants are exploring the mouth to help resolve uncertainty or confusion about the unfamiliar language input (Lewkowicz and Hansen-Tift, 2012). How could this explanation apply to the present findings in the case of sign language? Perhaps the 11-month-old infants tested here were also being presented with an *unfamiliar* visual language; hence, they look to the *mouth*. Importantly, 6-month-olds, however, look for *articulators*, and in the absence of movement on the mouth, they find it in the signing space that contains hands, while 11-month-olds look to the mouth because, for them, the mouth is their primary mode of articulation. This suggests an initial openness to explore possible articulators in multiple language modalities that attunes with age.

Several limitations need to be overcome in future work. First, it is worth mentioning the caveat common to infant perceptual studies. As with any study measuring looking behavior in infants, that an absence of differences in overt gaze across stimulus types reflects the absence of underlying sensitivity needs to be taken with a grain of salt. Of course, equal looking preferences or attention patterns may still result even if they can tell the difference between any two stimuli.

A second limitation is that we did not obtain concurrent measures of language development. We also did not obtain measures of stimulus comprehension in the native signing children. An important need to be addressed in future studies is the addition of visual attention measures, as in the present study, with concurrent and prospective measures of sign language outcomes (for discussion, see Henner et al., 2018). Measures of visual attention with overt gaze and eye movements do reflect underlying sign language proficiency in children (Lieberman et al., 2015, 2018; MacDonald et al., 2018, 2020). Gaze metrics have important utility because there is now substantial evidence that selective attention to language cues in the environment is tightly correlated with later social and language developmental outcomes (Tenenbaum et al., 2015; Tsang et al., 2018; Morin-Lessard et al., 2019). Moreover, the development of perception of body actions is important to study because this skill is one of the first prerequisite steps that support growing complexity in later expressive language skills and social development (Paulus et al., 2013). Another important consideration for future work is that children’s experience of gesture varies across cultures, families, and individuals (Kendon, 2004) in a way that can impact young learners’ perception of body actions.

## Conclusion

In the first year of life, infants actively attend to language cues, both visual and acoustic, in their environment and improve their perceptual abilities to recognize, discriminate, and categorize relevant language signals. Over time, the home language input changes their attention to these signals. Our study complements past findings, including those of infants’ attention to the speaker’s face, but also challenges interpretations to be broadened, as this body of research is typically framed in the context of speech processing. We found evidence that infants search for relevant linguistic information in either visual or auditory modalities. These results extend our understanding of infants’ set of tools use for learning language; infants are guided to look for language signals in both the sign and speech.

Finally, it is worth noting that the non-signing and signing infants and children tested in the present study are similar in that all have full language access since birth. The CODA children tested in the present study showed typical development that is appropriate for their visual language modality, reflected in a refinement in the visual attention for visual body actions, suggesting an acquisition of amodal pragmatic skills for communication. That is not the case for most deaf children who are raised by non-signing hearing parents. The majority of children born deaf have parents who hear normally and do not sign (Humphries et al., 2012; Hall, 2017). These infants may be missing critical learning strategies that native signers quickly acquire shortly after birth (Mayberry, 2010). Deaf children who are not exposed to ASL may not learn to use their “perceptual span” to gather linguistic information effectively. That hearing infants were attentive to sign language cues, even if sign language is not their home language, suggests that all infants are receptive to language as visual or manual.

## Author’s note

A video abstract of this article is available here: https://youtu.be/vQ8z5VDtxZs.

## Data availability statement

The raw data supporting the conclusions of this article will be made available by the authors, without undue reservation.

## Ethics statement

The studies involving human participants were reviewed and approved by University of California, San Diego. Written informed consent to participate in this study was provided by the participants’ legal guardian/next of kin. Written informed consent was obtained from the participants or minor(s)’ legal guardian/next of kin for the publication of any potentially identifiable images or data included in this article.

## Author contributions

RGB collected the data. RGB and DPC wrote the manuscript. All authors designed the experiments and contributed to data analysis and manuscript and approved the submitted version.

## Footnotes

1 Perceptual attunement has been used synonymously with “perceptual narrowing,” wherein infants lose sensitivity for non-native contrasts whilst gaining sensitivity for native contrasts between 8 and 12 months (Tees and Werker, 1984; Kuhl et al., 1992); however, we use attunement to refer to when perceptual sensitivities qualitatively change, perhaps becoming more or less specific, to fit or attune to the input.

2 There are certainly perceptual pressures to look directly at the hands, i.e., “articulators,” if one wants to perceive them clearly. First, the majority of signs have a specific location in front of the torso below the mouth (Sehyr et al., 2021). During signed conversations viewed a few feet away, the interlocuter’s hands primarily fall, on average, 6.5°, and as far as 16°, below the interlocuter’s eyes (Bosworth et al., 2019). Therefore, the hands, when fixating on the face, are seen with a spatial resolution of ∼25% of that seen with foveal vision (Henderson, 2003).

3 Recall that all signs and body actions (Grooming and Mimes) were executed by the native signer model starting and ending with her hands at “resting” at her sides. These movements are typically considered to be “transitional” movements. The designation of path and no-path movement refers to the articulation of the sign and body action tokens that were executed in between this transitional movement envelope. Movement paths were considered to be those sign or body action tokens with a clear ballistic motion of the arms and hands, while no-movement path designated forms that consisted of a relatively fixed contact point on the body.

4 Specifically, we counted frames when the hands were in the torso space for our stimuli which was about 75% of the time. This estimate agrees with what is generally known about signing space (Bosworth et al., 2019; Sehyr et al., 2021). Even for signs that have contact with the head, which comprise about 20% of lexical signs in ASL, the hands must go through articulatory space.

5 One could ask if the hands fall to the sides of the signer and whether participants looked at them. The hands did fall in these regions about 3% of the time (based on frame count). There is an intriguing but small, right-side looking bias for both infant age groups and for all body action types. The combined Left and Right side AOI average looking values (not shown in figures) are as follows: *6-month-olds:* Grooming: 1.21%; Mimes: 2.25%; Signs: 0.49%; *11-month-olds:* Grooming: 0.24%; Mimes: 0.48%; Signs: 0.55%. The Left and Right side AOI average looking values for the young children in Experiment 2 were less than 1%.

6 In this line of reasoning, pantomime is “more” symbolic than grooming, because pantomime involves acting on a non-present object, while for grooming actions, the body is present. Take “turning key” or “moving computer mouse” mimic actions; it is unlikely that infants have experience with these. Nonetheless, both require understanding the actor’s intent.
