# Supplementary material for: Visual attention for linguistic and non-linguistic body actions in non-signing and native signing children
Source: Front Psychol. 2022 Sep 9;13:951057. doi: 10.3389/fpsyg.2022.951057 (PMC9505519; doi:10.3389/fpsyg.2022.951057)
Supplement: Supplementary file 1 [file Data_Sheet_1.docx]

**Supplementary Materials**

1. Example videos of stimuli:
   1. [Examples of Signs](https://figshare.com/s/80ad776eaebb99798b9b)
   2. [Examples of Grooming](https://figshare.com/s/353391fe573286152353)
   3. [Examples of Mime](https://figshare.com/s/0c428b5560efc525c9e6)

**Table 1-A. English gloss of the ASL signs and body action gestures used in this study.**

| **Condition** | **Order in Trial** | **Body Action** | **Handedness** | **Handshape Change** |
| --- | --- | --- | --- | --- |
| Signs | 1 | ACQUISITION | 2 | change |
| Signs | 2 | SWEET | 1 | change |
| Signs | 3 | MEAN | 2 | change |
| Signs | 4 | PASS-OUT | 2 | change |
| Signs | 5 | INCOME-EARN | 2 | change |
| Signs | 6 | DREAM-DREAMING | 2 | change |
| Signs | 7 | ANNUAL | 2 | change |
| Signs | 1 | MELT | 2 | change |
| Signs | 2 | UGLY | 1 | change |
| Signs | 3 | EXAM | 2 | change |
| Signs | 4 | ASK | 1 | change |
| Signs | 5 | HATE | 2 | change |
| Signs | 6 | ABRUPT | 2 | change |
| Signs | 7 | LOCK | 2 | change |
| Signs | 1 | DOG | 1 | same |
| Signs | 2 | YELL | 1 | same |
| Signs | 3 | REQUEST | 2 | same |
| Signs | 4 | HEAVEN | 2 | same |
| Signs | 5 | FREE | 2 | same |
| Signs | 6 | HOT | 1 | same |
| Signs | 7 | FALL-IN-LOVE | 2 | same |
| Signs | 1 | CANCEL | 2 | same |
| Signs | 2 | BUILD | 2 | same |
| Signs | 3 | ABSURD | 1 | same |
| Signs | 4 | SCHEDULE | 2 | same |
| Signs | 5 | OCEAN | 2 | same |
| Signs | 6 | MATH | 2 | same |
| Signs | 7 | ELEPHANT | 1 | same |
| Mime | 1 | car - put key in and start ignition | 2 | change |
| Mime | 2 | throw basketball | 2 | change |
| Mime | 3 | tie a ribbon with both hands | 2 | change |
| Mime | 4 | catch a basketball | 2 | change |
| Mime | 5 | turn newspaper pages right to left | 2 | change |
| Mime | 6 | using a screwdriver | 2 | change |
| Mime | 7 | juggle two balls | 2 | change |
| Mime | 1 | move and click a computer mouse | 1 | same |
| Mime | 2 | paint a large wall in front | 1 | same |
| Mime | 3 | crack an egg with two hands | 2 | same |
| Mime | 4 | fanning face with one hand | 1 | same |
| Mime | 5 | wipe or clean windows | 1 | same |
| Mime | 6 | pick an apple put into basket | 2 | same |
| Mime | 7 | smooth wallpaper or a poster | 2 | same |
| Grooming | 1 | rub lotion on both hands | 2 | change |
| Grooming | 2 | dust off a shoulder | 1 | change |
| Grooming | 3 | crack knuckles | 2 | change |
| Grooming | 4 | scratch one ear | 1 | change |
| Grooming | 5 | pick at or clean one's nails | 2 | change |
| Grooming | 6 | rub and massage one's wrist | 2 | change |
| Grooming | 7 | stretch arms and hands | 2 | change |
| Grooming | 1 | adjust and pull down one's shirt | 2 | same |
| Grooming | 2 | rub both temples | 2 | same |
| Grooming | 3 | self-hug for warmth | 2 | same |
| Grooming | 4 | rub face | 1 | same |
| Grooming | 5 | pinch cheek | 1 | same |
| Grooming | 6 | finger through hair | 1 | same |
| Grooming | 7 | squeeze jaw | 1 | same |

**
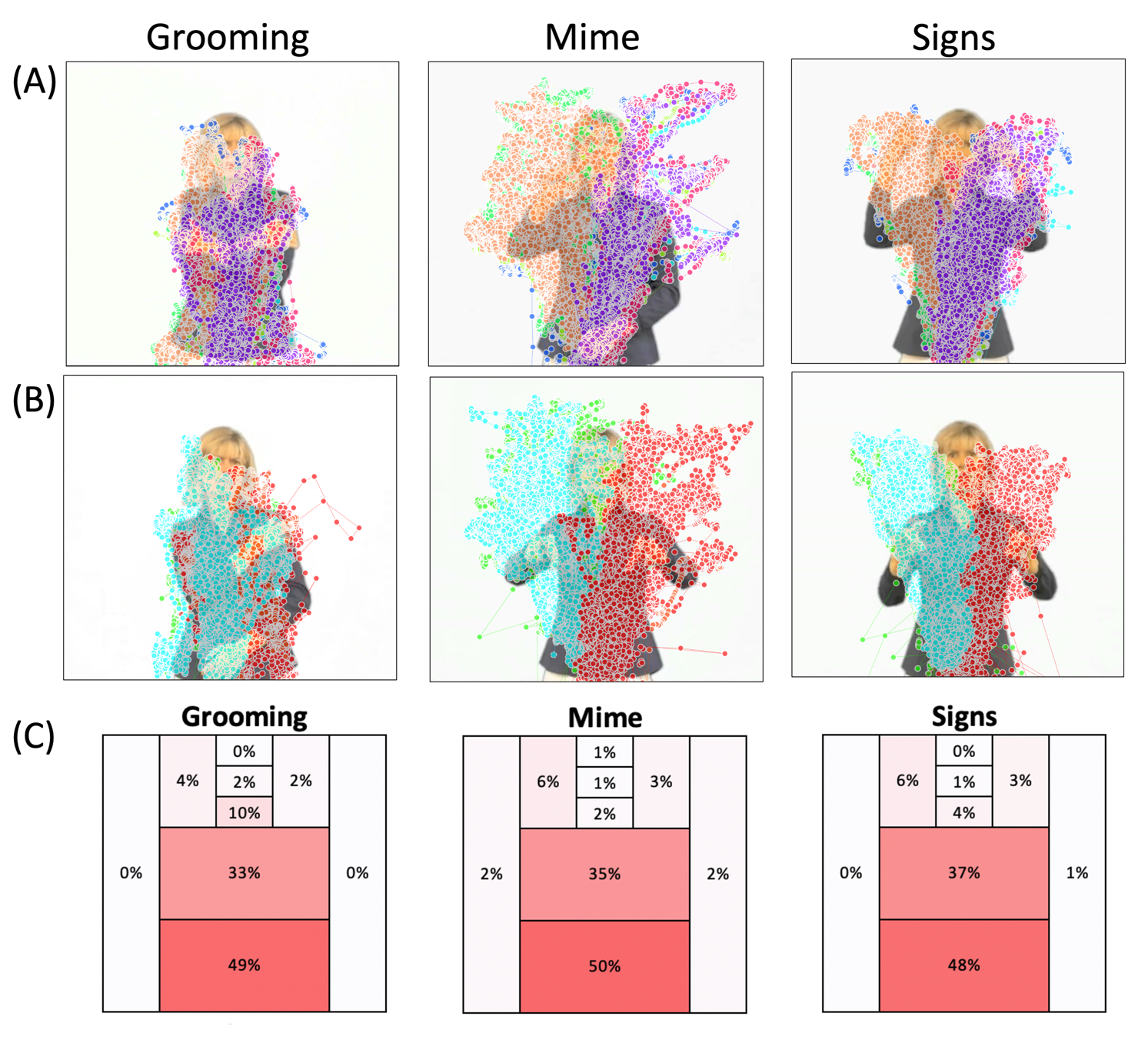
Figure 1-A. Occurrence of where the hands fall in space, measured using gaze patterns from three signing adult observers.** We asked three observers to track the left and right hand separately, three times, with the stimuli played at quarter speed. Presented here are gaze plots for two observers, (A) one highly fluent signer and (B) one novice signer. Observers were asked to track the center of the palm or knuckles (by necessity because sometimes the fingers were not visible, but this portion, either the back or front, was always visible). Sampling rate was 120 Hertz. (C) Average percent frame count of hand position, across the three body action types, the three observers, and left or right hand tracked. Each box indicates the average percent of time in which either hand fell. The hands fell most often on the belly, which is a by-product of our design because the signer was instructed to start and end there for each sign. The torso also represents an area where the hands fall mostly, during transition and during articulation.
